# The Value of Failure in Science: The Story of Grandmother Cells in Neuroscience

**DOI:** 10.3389/fnins.2019.01121

**Published:** 2019-10-24

**Authors:** Ann-Sophie Barwich

**Affiliations:** Department of History and Philosophy of Science and Medicine, Cognitive Science Program, Indiana University Bloomington, Bloomington, IN, United States

**Keywords:** philosophy of science, gnostic units, model pluralism, object recognition, history of science, localization, sparse coding, localist theory

## Abstract

The annals of science are filled with successes. Only in footnotes do we hear about the failures, the cul-de-sacs, and the forgotten ideas. Failure is how research advances. Yet it hardly features in theoretical perspectives on science. That is a mistake. Failures, whether clear-cut or ambiguous, are heuristically fruitful in their own right. Thinking about failure questions our measures of success, including the conceptual foundations of current practice, that can only be transient in an experimental context. This article advances the heuristics of failure analysis, meaning the explicit treatment of certain ideas or models as failures. The value of failures qua being a failure is illustrated with the example of grandmother cells; the contested idea of a hypothetical neuron that encodes a highly specific but complex stimulus, such as the image of one’s grandmother. Repeatedly evoked in popular science and maintained in textbooks, there is sufficient reason to critically review the theoretical and empirical background of this idea.

## Introduction: Why Talk About Failure in Science?

Science fails. It seems to fail at a high rate and with regularity. Experiments go wrong, measurements do not deliver the anticipated results, probes are contaminated, models are misleadingly simplistic or not representative, and some inappropriately applied techniques lead to false positives. One may wonder why science is so successful despite such prevalent failures. The alternative is to suggest that it is successful *because* of them.

Instead of a hindrance to scientific progress, a frequently overlooked positive characteristic of science is that it inevitably must fail to achieve an important job it sets out to do: discovery. For scientific research to exceed our initial modeling assumptions and to continuously supersede our ever-adjusting experimental limits, things have to go wrong. How science deals with failures may be more characteristic of the extraordinary nature of the scientific enterprise than its way of coping with successes.

It is one thing to say that science fails and that we should think about the decisive role of failures. It is another to highlight the particular aspects of failure that benefit our concrete dealing with science. Different attitudes toward science from the practitioner to the non-expert are the easiest to situate the lack of understanding of failure’s role in the scientific process. Such divergence of attitudes is apparent when it comes to the status of the scientific method. The scientific method is our most popular characterization and explanation for the success of science. But it has limits when it comes to reality. Being a traditional analytical tool and teaching device, it also paints a problematic picture of science because it never makes explicit the ubiquitous presence and influence of failures at the laboratory bench and modeling board (Medarwar, 1959/1999; Firestein, 2015; Schickore, in review).

This has several negative consequences of importance for theoretical perspectives on science. Intentionally or unintentionally it leaves laypeople with a distorted view of the changeable dynamics of empirical research; it perverts education policy and the methods of teaching science; moreover, it undermines the funding of science as a social contract. Indeed, one central danger coming from this lack of making the necessity of failure explicit is the increasing distance between the realities of scientific practice and its perception in the public and by funding bodies (O’Malley et al., 2009). Perhaps most pernicious, it further drives scientists to embrace a science that must prove its worth and restrict its inquisitiveness by counting its successes.

Not all of these problems will be solved by accepting failure as an inevitable, even required outcome of scientific practice. But all of these problems arise from our avoidance of failure. No solutions to these problems will present themselves until we first alter the view of failure as negative and better understand the precise role it plays in making science successful. One area where this can be accomplished with relative ease, and where the philosophy and history of science can serve an essential complementary role, is in our science narratives and analysis (Chang, 2004, 2012; Morgan and Norton, 2017; Schickore, 2018). These reconstructions are often in the form of “arcs of discovery” in which a long line of crucial discoveries seem to line up in perfect order and lead directly to fresh insight. But they are less than half of the story (Root-Bernstein, 1988, 1989). The failures, the errors, the cul-de-sacs, the long periods of being stumped, the “barking up the wrong tree” - all of these play a crucial role in revealing how science works, must work to produce the deep explanations that finally result (Firestein, 2012, 2015).

What makes failure in science theoretically interesting? Dealing with failures constitutes an active engagement with the limits of understanding. Successes primarily enforce current research strategies and models. Failures encourage us to broaden our perspectives on the nature of a particular problem and to open exploration to alternatives. Notably, this view amounts to more than merely saying ‘failure forces you to think of something new.’

Evaluating certain ideas explicitly as failures offers concrete guidance to a more interesting question, namely: *How* to think of something new? The heuristic function of failure in science can be illustrated with the case of “grandmother cells” in neuroscience. The idea of grandmother cells describes a hypothetical neuron which encodes and responds to a highly specific but complex stimulus, such as one’s grandmother (Barlow, 2009). Current neuroscience has not disproved but mainly forgotten about this idea, yet it occasionally resurfaces in popular science. What fresh insights into higher-level processing can the concept of grandmother cells offer when *analyzed as a failure*?

The article proceeds as follows. The next section introduces the historical origins of grandmother cells before breaking down the different lines of evidence and challenges involved in their investigation. Following this is a critical evaluation of why the idea of grandmother cells can be considered a failure, and how this treatment also informs theoretical perspectives on science, especially for the avoidance of relativism in the presence of model pluralism. The paper concludes with a broader outlook on how thinking about failure informs science, including science communication and funding.

## Grandmother Cells, an Almost Successful Idea

### Historical Origins

The term “grandmother cell” originated with the famous neuroscientist Jerome Lettvin. In 1969, during a lecture to students at MIT, Lettvin mockingly illustrated the hypothesis that complex concepts might have localized neural representations with an anecdote about a fictional Russian neurosurgeon, Akakhi Akakhevich. Akakhevich was called to see the protagonist of Philip Roth’s novel *Portnoy’s Complaint*. Portnoy had a troubled relationship with his mother, and Akakhevich treated him by identifying and removing those brain cells responsible for encoding Portnoy’s memory of his mother. Post operation, Akakhevich remarked that the patient comprehended the concept of “mother”, yet lost all associations with *his* mother. Lettvin mused that, encouraged by his success, Akakhevich moved on to the study of “grandmother cells” (Barlow, 1995, 2009).

Starting as a joke, the term spread quickly in the 1970s, periodically revived in later decades. Initially dismissed (Barlow, 1972), a couple of first experimental results lent the idea some plausibility (Perrett et al., 1982; Rolls, 1984; Yamane et al., 1988; Gross, 2002). What fostered appeal to the possible existence of grandmother cells?

Grandmother cells were an embodiment of the neuroscience *Zeitgeist* of the 1960s to ‘80s; their allure was their metaphorical potential. Of course, metaphors have a long tradition in scientific reasoning (Hesse, 1966; Bailer-Jones, 2002). Consider Dalton’s “billiard ball model” or Rutherford’s “solar system model” of the atom. These constituted powerful images, which brought current lines of empirical evidence under a theoretical umbrella. Similarly, the concept of grandmother cells encapsulated several prominent research hypotheses in early neuroscience as a burgeoning field (Shepherd, 2009). These hypotheses could be subsumed under the question of how, after initial sensory processing, information gets integrated at later stages in central processing (Hubel and Wiesel, 1979).

**FIGURE 1:**
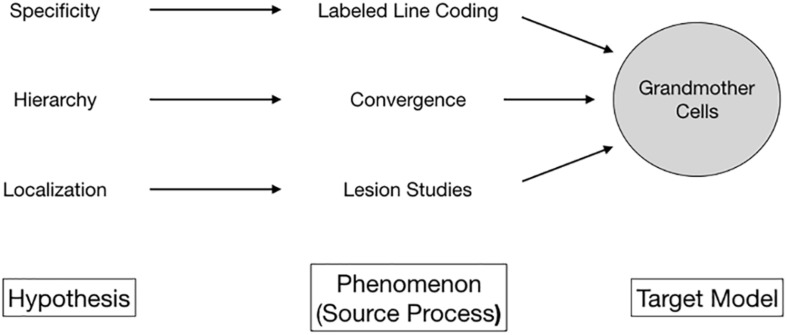
Multiple lines of empirical evidence line unified to a unified phenomenon by the conceptual proxy of grandmother cells.

What granted grandmother cells empirical plausibility? Three hypotheses stood out (Figure 1). First, it turned out that brain cells are choosy; they respond selectively only to specific kinds of input. Over the first half of the twentieth century, such specificity of neural responses was supported by emerging empirical evidence for *labeled line coding*, with neurons and anatomical areas responding selectively to specific stimuli (Adrian and Matthews, 1927). Second, evidence for selective neural activity soon was tied to the idea of *localization*, the assumption that highly specialized cells formed clusters and aggregated in some regions of the brain. Lesion studies by Karl H. Pribram and Mortimer Mishkin (Pribram and Mishkin, 1955; Mishkin, 1966), the discovery of cortical columns by Mountcastle (1957), and the cat striate cortex experiments by Hubel and Wiesel (1962, 1965) were at the core of this development. Although called into question in recent years, localization as a paradigm persists today (Burnston, 2016). Third, it transpired that stimulus processing involved the *hierarchical convergence* of neural signals. Basically, the higher-level the stage of processing in the brain, the more specialized its output. The visual pathway presented an excellent model for this idea (Marr, 1982; Hubel, 1988). Inference from cells coding for edges, shapes, and junctions facilitating stable object recognition (Oram and Perrett, 1994) to, eventually, object-specific cells – firing in the presence of tables and perhaps individual people – was at least at hand.

Implications surrounding the existence of grandmother cells proved tempting, with one question taking center stage: Could this concept resolve the fundamental issue of whether neural representations of objects, including the specialization of cell responses, were more or less hard-wired (Hubel and Wiesel, 1963) or learned (Blakemore and Cooper, 1970)? The psychologist Rose (1996, 881), who trained under Colin Blakemore, remarked: “The reason I thought ‘grandmother cell’ such as good term was that it implied that, if the theory were true, each of us would have different wiring for our grandmother cells; hence this wiring could not be innate.” The term had heuristic potential.

Evidence for such type of cell remained indirect, however. This was not a surprise. To be sure, the concept’s metaphorical origins did not fool scientists to hold strong ontological beliefs about the existence of literal grandmother cells. Rather, the persistence of this term in neuroscientific imagination sometimes mirrored a lack of credible alternatives, as Hubel and Wiesel (1979, 52) found: “What happens beyond the primary visual area, and how is the information on orientation exploited at later stages? Is one to imagine ultimately finding a cell that responds specifically to some very particular item? (Usually one’s grandmother is selected as the particular item, for reasons that escape us.) Our answer is that we doubt there is such a cell, but we have no good alternative to offer.”

### The Concept and Its Name, Demetaphored

The concept of grandmother cells began as a humorous anecdote. It soon became a metaphor for the research strategy of an expanding neuroscience before it participated in scientific discourse as a genuine term. Indeed we find the idea of grandmother cells still discussed in modern textbooks of cognitive neuroscience (Gazzaniga and Ivry, 2013), albeit with reference to its hypothetical character. What might account for such deceptive success?

**FIGURE 2:**
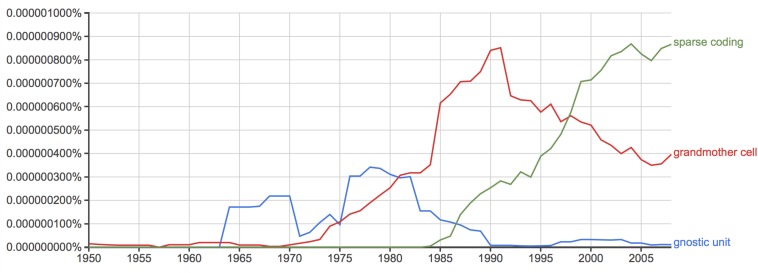
(Data source: Google NGram): Frequency comparison of the keywords “grandmother cell,” “gnostic unit,” and “sparse coding” in the years between 1950 and 2008.

The question needs reframing: Was the unlikely success of grandmother cells really an indicator of the general success of the field or, maybe, a sign of its stagnation? By the 1970s, and 80s, when the concept of grandmother cells took off (Gross, 2002; see also Figure 2), the revolutionary developments in mid-twentieth century neuroscience temporarily came to a halt. From a broader perspective, the vision scientist Marr (1982, 14) observed: “But somewhere underneath, something was going wrong. The initial discoveries of the 1950s and 1960s were not being followed by equally dramatic discoveries in the 1970s. No neurophysiologists had recorded new and clear high-level correlates of perception.”

Marr identified the lack of proper theory as a problem; answers about mind and brain could not be found on a single cell level, as champions of grandmother cells had suggested (Barlow, 1972), but required a framework of the general processes of neuronal signaling. Physiologists diligently measured single cell responses, without arriving at the bigger picture of signal coding that these recordings ought to convey. In response, in his landmark publication *Vision*, Marr (1982) proposed a computational understanding of perceptual processing that would invigorate and shape research in neuroscience in the following decades (Glennerster, 2007; Bickle, 2015). Meanwhile, the concept of grandmother cells seemed to encapsulate the stalled success of the assumptive framework of convergent, hierarchical, and localized signal integration – as a vague enough proxy for a conceptual space without proper theory or mechanistic explanation.

The legitimacy of the concept grounded in its epistemic accessibility and descriptive function. Complex processes of signal construction could be described as sequential, linear, and mirroring common sense about mental concepts representing real-world objects (such as grandmothers and tables). This descriptive appeal of grandmother cells stood in stark contrast with preliminary idealized computational frameworks like McCulloch and Pitts (1943) that count as early predecessors of contemporary neural network models (Piccinini, 2004). Signal coding in binary code did not necessitate a physical localization of cognitive objects in neural space. Still, thinking globally about the brain in terms of information processing was abstract and, as yet, also too detached from research on its physiological basis (Gefter, 2015). By contrast, grandmother cells carried an air of concrete physicality and experimental discoverability.

But what of explanation? Explanation differs from description in the sense that it targets the mechanisms, the causal basis, that underpins a phenomenon (Machamer et al., 2000; Craver, 2007). Now, it is not uncommon for scientific concepts to start as proxies or conceptual placeholders. Such proxies enable scientists to describe separately observed but potentially connected phenomena under an umbrella term, even though the existence of that entity is purely speculative at the time. The success of such proxies in an experimental context hinges on their empirical consolidation and degree of manipulability, meaning their progressive entrenchment in an experimental context that allows identifying particular functions and physical structures with specific observable effects. Simply put: when heuristic descriptions manifest as causal explanations (e.g., see the history of cell surface receptors in Barwich and Bschir, 2017).

Grandmother cells lacked such explanatory basis. One reason for this shortcoming was that it was unclear what grandmother cells actually were; specifically, to what processes they referred. Because a central problem with the term was its conceptual murkiness. That did not stop this term from being used as a popular metaphor: “The term is flippant, incomplete, and not quite accurate, but it is widely understood, and I shall stick with it.” (Barlow, 2009, 313).

As intuitive the metaphor of grandmother cells was to common sense, as ambiguous was its translation into a valid scientific term. Unlike other metaphors in science, the concept of grandmother cells was never successfully demetaphored; “demetaphorization” referring to the epistemic process by which a discourse community standardizes a term (Temmerman, 1995; English, 1998). Scientific terms are highly specific, and their application tailored to a modeling task and target system. (Such specificity does not conflict with scientific concepts undergoing conceptual change or cross-domain variation; Barnes, 1982; Temmerman, 2000; Barwich, 2013.) Grandmother cells were opaque both on a theoretical as well as a practical level, which severely impeded their assessment.

From an empirical viewpoint, it was undetermined what kind of information or signal was processed and integrated with such a hypothetical neuron. For example, did grandmother cells only respond to visual input, or did they also process cross-modal cues, including auditory and olfactory signals? (After all, many people find that odors vividly connect to their memory of places and people; for example, the smell of one’s grandmother’s house; the aroma of her home-cooked food; and perhaps her perfume.) It was unclear whether (and if so where) there is such centrum of learned, cross-modal integration. In one way of another, this puzzle mirrored a broader challenge in research on signal integration that is generally discussed as the “binding problem” in cognitive neuroscience (Treisman, 1996; Roskies, 1999; Holcombe, 2009): How does unified phenomenal experience arise from separated and specialized neural processes? Besides, how stable or flexible was such hypothetical neuron in recognizing an individual entity, such as one’s grandmother, as being the same entity over time. If we haven’t seen someone in years, even decades, we often can pick out that person (in real life but also in photographs). What information or features grandmother cells were picking up was far from obvious.

From a theoretical perspective, the scope of the concept of grandmother cells was not clear-cut either. If describing an actual neuron, would grandmother cells respond only to a particular individual (*my* grandmother), or to several entities falling under the same category (*a* grandmother)? The origins in Lettvin’s story suggest the former. However, these fictional origins were largely unknown before the mid-nineties (Barlow, 1995) and, in practice, the two meanings were not always separated but frequently mentioned alongside each other (Gross, 2002; Barlow, 2009; Plaut and McClelland, 2010).

This was no coincidence, since the idea of concept-specific cells lured in the background. The notion of concept-specific cells was proposed, somewhat independently, at the same time as Lettvin’s grandmother cells. Sealed off behind the Iron Curtain, the Polish neurophysiologist Konorski (1967) had advanced the idea of “gnostic units,” forming “gnostic fields.” Konorski’s gnostic units soon were associated with recordings of localized responses to complex objects, such as faces (Perrett et al., 1987). And more experimental evidence emerged for object-specific activity in neural domains, involved in the perception of objects or categories such as hands, locations, or emotional expressions (Gross, 1998). These findings further resonated with the broader philosophical tendency in favor of the modularity of cognition and its neural underpinnings (Fodor, 1985).

Perhaps its theoretical ambiguity granted the concept of grandmother cells some momentum with empirical support, since both concepts, grandmother cells and gnostic units, seemed sufficiently similar. However, they are not.

It is important to keep these two concepts apart because they are not co-extensive in their application and do not imply the same processes: “if we were persuaded that grandmother cells do not exist, that would not be a reason for concluding that localist cells do not exist” (Coltheart, 2016). Recordings of localized responses can result from different mechanisms of information encoding. Conceptual blending of grandmother with gnostic cells thus could filter out relevant distinctions that may suggest another explanation via a different kind of mechanism (see section “Making Other Explanations Explicit: From Grandmother Cells to Sparse Coding”).

Curiously then, the difficulty with grandmother cells lies not in their metaphorical framing or descriptive content *per se*, but how this concept influences the explanatory structure of arguments on signal coding. On this account, we will see next that the failure that characterizes the idea of grandmother cells is not its lack of direct experimental support or any form of apparent falsification. Many scientific concepts survive, even thrive, despite prolonged absence of empirical support. Instead, it is the neutralization of significant distinctions in causal explanations that would have implied and strengthened “unconceived alternatives” (a term by Stanford, 2006).

## Failure Analysis Advances Pluralism Without Relativism

Should grandmother cells be considered a failure? The answer is not straightforward. That is precisely why this concept makes for an illustrative case. Grandmother cells are not an indisputable failure if we center our philosophical perspective on the justification of epistemic norms that undergird scientific practice, including the benefits of dissent (e.g., see debates in social epistemology; Longino, 2019). Indeed grandmother cells might be considered to represent a perfectly legitimate idea if we adopt a pluralist perspective. As an example, the philosopher and historian of science Chang (2012) provided a convincing argument about the Chemical Revolution: why it would have been beneficial to keep the old chemical principle of phlogiston alive next to the new chemistry of oxygen (in short: because it pointed toward observations that later were associated with electrochemical phenomena and thus may have accelerated these findings).

So why speak of failure then?

### Failure Meets Pluralism at the Dilemma of Choice

Hardly anything in scientific practice constitutes an obvious failure, except (and even) in hindsight. A look at the history of science tells us that many concepts we consider as failed today were never explicitly disproven. Routinely, these concepts merely faded in their relevance or heuristic power, replaced by alternative explanations. Disproving speculative ideas is not as easy a job as it sounds. Negative results need not be conclusive proof against a model.

Failure in scientific practice is a much more ambiguous notion than traditional ideas of falsifiability or null hypotheses. Schickore, a philosopher and historian of science, for instance distinguished between four interpretations of failure: omission; breakdown; not meeting one’s goal; and personal failure. Two kinds of failure are instructive in a scientific and educational context: (1) the “failure to follow a known and generally acknowledged rule or to miss a specific goal or target,” and (2) going amiss as the “stories of struggle, setbacks, and stagnation” which “by contrast, are about scientists finding themselves in situations where nobody knows what to do and what is right” (Schickore, in review). The second type of failure is less clear but permeates everyday science beyond the benefits of historical wisdom. Moreover, it is not only less evident as a failure, but also has no appropriate rule by which to proceed or change course.

The next section presents the story of grandmother cells as an expression of the second type of failure. On this account, we must wonder what determines the threshold of reasonable doubt to find that an idea may have turned into a dead end, and how to conceive of reasonable alternatives. Surely, some ideas are dead ends, and it matters to lay such ideas to rest because their faux success might obscure inquiry into better-suited, more fruitful lines of investigation. Moreover, the false appearance of success of some ideas can seriously distort theoretical perspectives on the advancement and objectives of an experimental system. A case in point is the debate surrounding the pseudo-controversy of the “quantum nose” (Barwich, 2018a); here, a misconception of what constitutes the empirical success of a model led to a fundamental gap between specialist and non-practitioner views on evidence, including a field’s progression. Such concerns should matter if we aim to advance theoretical perspectives, such as in the philosophy of science, as complementary to science (Chang, 2004) or as “explication work”: including the philosophical “analysis of how scientific concepts and practices have developed,” and “the explication and clarification of our analytic tools” in science studies (Schickore, 2018, 19 and 16). This is why we need to talk about failure in science.

From this perspective, focusing on the notion of failure first sounds like a challenge to pluralism. Scientific pluralism is the philosophical view that science is most progressive when it maintains and works with various, sometimes even conflicting models and methods (Kellert et al., 2006). Speaking of failure seems to imply the opposite: that we drop concepts that are considered false or are in dire conflict with other models. However, this opposition is misleading.

Pluralism and failure meet at the dilemma of choice. A general objection directed at scientific pluralism is its abundance of options, which opens up concerns of relativism, as Chang (2012, 261) observed: “The fear of relativism, and its conflation with pluralism, will not go away easily. The objection comes back, in a different guise: ‘If you go with pluralism, how do you choose what to believe?’ Well, how *do* you choose in any case?” (emphasis in original) Pluralism does not preclude choice but suggests that choices are not exclusive, whether such decisions concern the selection of questions, methods, models, or hypotheses. Failure analysis aids in identifying and making such choices.

### Making Other Explanations Explicit: From Grandmother Cells to Sparse Coding

This is where we get back to our case study. Effectively, grandmother cells never became a success. Hubel (1988) explicitly rejected the notion, as did others. But the idea did not simply die either. While some neuroscientists suggested that it is about time to lay this concept to rest (Sejnowski, 2014), attempts to recover grandmother cells appealed to their plausibility (Bowers, 2009). Yet, reasoning from empirical possibility is too weak a criterion of success and, in the case of grandmother cells, not as robust as Bowers suggests (Plaut and McClelland, 2010).

What fueled the resurrection of grandmother cells? In the past couple of decades, the idea received regular revival in popular science; you may have heard about the so-called “Halle Berry neuron” or “Jennifer Aniston cell” (e.g., Gosline, 2005; Khamsi, 2005; Gaschler, 2006). These reports cover the discovery of single neurons in humans that respond selectively to various visual representations of individual people (i.e., different images of a person, but also their written names), as well as other highly specific objects, including iconic places such as the Sydney Opera House (Quiroga et al., 2005, 2008, 2009).

Could these neurons be the mythical grandmother cells? Science writers quickly made the connection. Yet the more cautious among them begged to differ: “Quian Quiroga does not, however, believe these neurons are grandmother cells, at least as they were initially conceived” (Zimmer, 2009).

Quiroga et al.’s recordings showed a number of cells that indeed responded to particular individual entities in a strikingly selective manner. However, a closer look at these findings suggests a substantially different model of stimulus coding and memorization than that in support of grandmother cells. Specifically, as the authors themselves emphasized – upfront in one of the publication titles – their discovery revealed *Sparse but not ‘Grandmother-cell’ coding in the medial temporal lobe* (Quiroga et al., 2008). Drawing that difference is crucial.

Specialized responses to particular objects such as faces or hands have more recently been explained in terms of *sparse coding* (Quiroga et al., 2013). Sparse coding denotes the effective activation of a small group of neurons. In contrast with grandmother cells, and instead of a particular cell, the process of sparse coding implies *pattern activity* measurable in temporal and spatial dimensions, as well as firing strength. Notably, the hypothesis of sparse coding has taken over prevalence in comparison to grandmother cells over the past twenty years (Figure 2). What constitutes the difference in coding between grandmother cells and sparse coding?

One significant difference is that sparse coding is not exclusive. Neurons might fire selectively in response to highly specific stimuli, such as Halle Berry. But this is not the only stimulus to which they respond. Additionally, sparse coding does not show the encoding of particular stimuli as separate or isolated entities. Instead, it builds a *network of associations* between familiar items: While a limited number of neurons responded to specific stimuli (say, pictures of Jennifer Aniston), these cells also responded selectively to specific stimuli known from the same context (namely, Lisa Kudrow, the actress starring next to Aniston in the sitcom *Friends*). Alternatively, should we think of sparse coding in terms of concept cells or gnostic units then?

At this point, the earlier suggested difference between grandmother cells and gnostic units comes into play. The model of sparse coding seems to resonate with Konorski’s idea of gnostic units. Notably, Konorski (1967) proposal was markedly different from grandmother cells. To show this, we first need to separate the mechanistic explanation of “concept cells” in terms of sparse coding from the descriptive function of the grandmother cell idea. The descriptive function of grandmother cells was its role as a proxy in a linear model of hierarchical object coding, where simpler signals turned into higher-level representations via cells responding to ever more complex signals (Hubel, 1988; Gazzaniga and Ivry, 2013). In comparison, sparse coding can but need not imply linear or hierarchical stimulus encoding; it is not co-extensive with the principles of input-driven object recognition.

In fact, sparse coding indicates *an entirely different theory of neural representation*. Sets of neurons build a net of learned associations, potentially unrelated in their feature-coding pathways (e.g., information from edge detection merges with auditory input). These sets do not code concepts strictly bottom-up, from simple signals to complex individuals or categories. Rather, stimulus representation might be governed by multiple organizational principles, including top-down effects and statistical frequency next to bottom-up coding.

Three observations exemplify the difference. First, the cell that responded selectively to Bill Clinton did not fire in response to images of other United States presidents (Quiroga et al., 2005). So these cells were not simply neural representations of semantic categories and conceptual clusters. Second, when faced with ambiguous stimuli, such as the image of Bill Clinton morphing into George Bush, neural responses mirrored the subjective reports of the participant. In other words, the activity of these neurons did not correlate with stimulus similarity, but the subjective perceptual judgment about stimulus similarity by the participants (Quiroga et al., 2014). Third, unrelated stimuli presented in close temporal proximity could become associated under a familiar concept, such as verbal with visual cues like names with faces (Reddy and Thorpe, 2014). This indicates a substantially different causal mechanism in the formation of concept cells than the idea of grandmother cells.

The new line of inquiry emerging from these developments is: What are the *association mechanisms* that govern an individual’s learning of similarity as familiarity relations between highly specific stimuli and, further, their encoding into long-term memory under particular categories? An answer to this question requires a theoretical framework different from the reasoning that fueled the idea and plausibility of grandmother cells.

To conclude this point, grandmother cells and sparse coding share one central hypothesis; localization. But localization is not self-explanatory and can be an expression of various mechanisms of stimulus encoding (Coltheart, 2016). The crucial difference between grandmother cells and sparse coding thus is that the idea of grandmother cells emerged as a direct consequence of the hierarchical coding hypothesis. In comparison, sparse coding does not necessitate hierarchical coding; it primarily builds on a theory of learning, especially associative learning (Reddy and Thorpe, 2014).

### (Why) This Idea Won’t Simply Die

Despite these recent developments in research on alternative coding schemes, the idea of grandmother cells continues to attract sporadic interest. In particular, Roy (2012, 2013, 2017) published a series of opinion pieces and a conceptual analysis defending the idea based on his proposal of a localist theory of abstraction in the brain. This defense is noteworthy to complete our failure analysis for two reasons. First, it exemplifies an implicit disciplinary schism in explanations of the brain. Second, it shows that the issues that challenge the idea of grandmother cells are not of an isolated scientific concept, but of a broader understanding of the brain. That is why the idea of grandmother cells won’t simply die.

Data never just speaks for itself and no scientific concept ever makes sense without its supportive framework. In neuroscience, this mirrors an enhanced dilemma, as one reviewer pointed out. Because research on the brain tends to be divided into two approaches: On the one hand, there is the computational, more abstract mechanistic style of analysis in cognitive psychology and related disciplines interested in modeling information theoretic systems. On the other hand, there is direct research on biological, molecular and cellular mechanisms. The essential tension, frequently separating these two perspectives, is that computational models can remain divorced from biological reality. Meanwhile, neuroscientific findings can lack proper theoretical grounding – so much so that the nature of the data, as determined by technology, often “masquerades as explanation” (an expression I owe to said reviewer). This problem is illustrated by many methodological debates; for example, the dilemma of reverse inference in neuroimaging, such as fMRI analysis (Poldrack, 2006, 2011).

Are some interpretations of the data promoted by inherited yet debatable conceptual intuitions? What empirical issues are left out that require proper explanation while being missed by not subjecting certain philosophical preferences to failure analysis? One such oversight concerns the mechanism of categorization as information clustering; namely, how the brain acquires and develops its information-specific activity. The previous section indeed has shown that more than one explanation is possible. It foregrounded an alternative where category-selective cellular activity builds on association mechanisms (implemented via sparse coding), instead of the hierarchical coding scheme endorsed with the concept of grandmother cells.

Roy’s (2012, 2013, 2017) argument for grandmother cells, as a special case of a semantic localist framework, is a good example to highlight the divergence in assumptions here. Roy’s (2013, 1) definition of grandmother cells is a follows: “a grandmother cell represents a specific and complex concept, not merely a percept, in a multimodal invariant way. Thus, the basic grandmother cell notion is about encoding a complex concept within a single cell in an invariant way. And abstract categories—such as animals, cars, and houses—are, without question, complex concepts. Category-type concepts, therefore, are part of (or included in) the notion of grandmother cells. (…) In a more general sense, grandmother cells are fundamentally about abstraction and generalization.” Roy’s argument for the existence of grandmother cells involves four claims: (1) Cortical columns (clusters of cells with similar receptive fields) are the fundamental functional and computational unit in the neocortex. (2) There is localized object- and category-selective cell activity. (3) The nature of information processing in the brain is abstraction. In support of this are recordings from modality-invariant cells. And (4) on neural representation: there is no evidence for dense distributed representation, population coding is too slow for fast neural decision-making processes, and sparse coding is only used in the encoding of memories. This leads Roy to claim, “at an abstract level, the brain is a massively parallel, distributed computing system that is symbolic.”

The biological foundation of neural processing does not square with this conceptual design of the brain. Specifically, the (1) functional significance of cortical columns, as an anatomical and developmental structure, has been called into question (e.g., Horton and Adams, 2005). Besides, (2) neither modality invariant nor category-selective cell activity is sufficient to indicate single-cell over sparse encoding of information, as the previous section illustrated. Further, the conflation of grandmother with concept cells in Roy potentially obscures different coding and association processes; how the brain identifies individual entities (*my* car) may not reside in processes identical to categorization (*a* car). (3) Whether abstraction defines the ultimate nature of the brain is debatable; and even if one pursues an abstraction-centered framework of the brain, this premise is independent of localist or non-localist models of a specific flavor. [Roy’s adopted computational understanding of the cognition as symbol processing rests on Newell and Simon (1976), who defined cognitive processing in computational terms, not its instantiation in the brain. Such symbolic, representational account is far from uncontested (e.g., McClelland et al., 1995; Van Gelder, 1995)]. Lastly, (4) there is plenty of evidence for distributed representation and population coding in electrophysiological research that counters Roy’s description. Over the past two decades, these findings, in invertebrates but also vertebrates, resulted in experimentally driven, highly sophisticated information processing models addressing temporal dynamics and the encoding of multidimensional stimuli, including non-visual information (e.g., Laurent, 2002).

Overall, there is sufficient experimental reason to question not just grandmother cells but the implicit, overarching style of reasoning in traditional localist theories of the brain (Burnston, 2016), or treating its function exclusively in terms of semantic or language-based symbolic abstraction and representation (Keijzer, 2001; Chemero, 2009). Indeed a central problem that has plagued more recent approaches to reviving the idea of grandmother cells is the deeply problematic use of ad hoc hypotheses (Grünbaum, 1976); meaning the attempt to redefine a notion into existence by repeatedly adjusting its definition in light of newer observations. What makes this practice problematic is that it sidelines notable observations that do not square with such definitional exercise. A case in point is Quiroga et al. (2014) finding that, when faced with ambiguous “concept” or “person” stimuli, neural responses mirrored the subjective reports of the participant. This top-down, decision-making component in stimulus-selective cell responses gets buried in frameworks that want to fix the fundamentals of neural coding by stimulus-specific single-cell responses.

But the critical point here is the mechanism of information coding. What *kind of* computational mechanism undergirds categorization and illustrates how the brain establishes neural linkage in information processing? The previous section had shown that the category-selective responses of cells could be explained by associative learning via sparse coding. Roy’s explicit rejection of this explanation resides in the claim that sparse coding does not facilitate generalizations. A claim that is drawing on his reading of McClelland et al. (1995), who “have argued that sparse distributed representation doesn’t generalize very well and that the brain uses it mainly for episodic memories in the hippocampus” (Roy, 2017, 3). A closer look at McClelland et al.’s original paper yields a different understanding. The issue here is not the possibility of *generalization per se* (as the process of categorization, including the learning of concepts and recognition of examples), but the specific way conceptual inference and classification is established and learned.

Basically, the decisive question is: Do we recognize (new) instances or stimuli as belonging to a specific category based on hierarchical semantic links, “as a short form of the statement *An X is a Y*” (McClelland et al., 1995, 428)? Or do we build generalizations via “the discovery of shared structure through interleaved learning” (ibid.) McClelland et al. argue for the latter on the basis of a connectionist model, involving sparse coding, that offers an explanation of how associative learning facilitates categorization. Such computational framework resonates with the explanation advanced in the previous section, further eroding the philosophical intuitions in favor of a hierarchical semantic network (that had fueled appeals to grandmother cells). McClelland et al. (1995, 428) had noted about the latter: “Semantic networks of this type were very popular vehicles for representation for a period of time in the 1970s, but apparent experimental support (Collins and Quillian, 1969) for the hypothesis that people’s knowledge of concepts is organized in this way was illusionary (Rips et al., 1973).”

Such a difference in network modeling and the diverging understanding of computational processing in the brain furthers the conclusions in the previous sections: that failure analysis helps us to identify and make *conceptual choices* in the interpretation of experimental data. These choices, we saw now, also concern the dilemma of how data really links with theory, and vice versa, exemplified by the challenge of how to connect highly theoretical models with detailed neurobiological evidence. If we start *analyzing* some concepts explicitly as failures, *how* does this advance our understanding of a subject matter – such as information processing in the brain with examples like grandmother cells?

### Unconceived Hypotheses: Background Cells?

There are multiple reasons why the idea of grandmother cells, including the framework on which it rests, is ripe for failure analysis. The plausibility of grandmother cells boiled down to their descriptive function as a *conceptual proxy* (lacking a proper mechanism) in a widely successful, associated framework; namely, input driven, hierarchical coding in object recognition. This framework remains prevalent to date. But, compared to the 1970s, for different reasons today.

Some deep learning trials and teaching resources continue to work with the principal idea of grandmother cell coding (e.g., Convolutional Neural Network or CNN) – which further exemplifies the difference between the aims of computational modeling and research that targets the real brain. While coding schemes for grandmother cells are certainly possible to implement in neural networks, and may be used in technological applications, that procedure may not tell you about how the brain works. In wet-lab neuroscience, grandmother cells hardly find mention (perhaps with the exception of Barlow (2009), who held on to his original neuron doctrine from 1972, involving single-cell coding). Meanwhile, the broader change in understanding the mechanisms that undergird object categorization and representation in neuroscience happened with the increasing ability to record from multiple neurons and neural populations (Sejnowski, 2014).

In addition, the monopoly of strictly input driven, hierarchical coding has been called into question on several other grounds: e.g., its underlying non-contextual localization paradigm (Burnston, 2016); its visuocentric and input-driven model of stimulus representation (Barwich, 2018b); as well as increasing evidence for the stark influence of experience on the formation and maintenance of category-specific neural domains, such as the fusiform face area (Livingstone et al., 2017). While such concerns may not debunk traditional views of object recognition, they do recommend revision. Lastly, the idea of grandmother cells also mirrored central problems inherent in this broader framework, like the “binding problem” (section “The Concept and Its Name, Demetaphored”), which could be explained by alternative models such as Prediction Error Minimization in more recent proposals surrounding the predictive brain (Hohwy, 2013). In light of this, sticking with grandmother cells may not be our best way forward.

Failure analysis, meaning the treatment of a concept explicitly as a failure, can aid to identify and make choices regarding our modeling assumptions in this context. Subjecting the idea of grandmother cells to failure analysis means questioning its implicit reasoning in order to develop alternative views. For this, we need to carve out the conceptual choices that this reasoning entails. Failure analysis, so understood, is primarily a method, not an ontological commitment. It does not even preclude the future discovery of grandmother cells. But it does make clear the costs of keeping a withering concept alive. The notion of grandmother cells was all about stimulus-induced, foreground object formation. This sidelines other, equally essential lines of inquiry.

Object recognition, under natural conditions, is part and parcel with the distinction of environmental background. Focus on the computational principles that underlie background rather than object representation may foster significant discoveries. Rose (1996, 884) highlighted the importance of this kind of alternative inquiry: “It was Michael Arbib (…) who introduced the analogy between vision and making an animated cartoon: first a cel (a transparent sheet of celluloid) is laid down on which the background is drawn; the sky, ground, clouds, trees and so on. Then other cels are overlaid on which are drawn objects closer and closer to the foreground: particularly characters and other objects that move, in other words that change from frame to frame. This is not only logical but also makes it easier to draw, in that the same background can remain in place throughout the scene, and only the foreground cels have to be redrawn.”

Should we assume the existence of “background cells”? Surely not. However, background computation and object recognition build upon the same perceptual processes. Rose (1996, 884) continued: “In neural terms, we know that keeping track of an animal’s location within its environment is, perhaps, one function of the hippocampus (…). This system keeps a record of what should be present in the environment, at least in a familiar environment such as the animal’s home territory. Against this background it is easy to detect any deviation from the expected pattern of incoming stimulation, in other words to detect novelty or change, to generate an arousal response and an orienting reaction that directs attention to the novel stimulus.” Rose’s suggestion here appears complementary to the reasoning that drove Koch and others to think about sparse coding: “Thanks to highly specialized cells, we recognize our own grandmother immediately in the crowd of other elderly ladies at the senior citizen home, without having to think twice about it” (Koch quoted in Gaschler, 2006, 82). We can memorize specific objects by their idiosyncratic features; recognizing those requires their differentiation from background noise and variation. Both background formation and object recognition build on statistical learning and stimulus frequency; processes that have taken center stage in current models in cognitive neuroscience.

In sum, the reasoning embodied by grandmother cells sidelined fruitful lines of inquiry. While picked up by contemporary research, these lines were already present in the 1970s (e.g., in Neisser, 1976). Such “unconceived alternatives” resonate with a phenomenon that the philosopher Stanford (2006) found in the history of science: namely, that many notable scientific ideas could have “made it” much earlier because their delayed success was not due to an absence of data, but poverty in conceiving the salience in evidence of alternatives. From this perspective, failure analysis acts as a conceptual tool of model pluralism without relativism: by making epistemic choices explicit and to question the plausibility of our more widespread, although implicit theoretical foundations.

## Conclusion: Failure Analysis

Failure in science has many flavors. This article offered one way to use failure as an epistemic tool propeling scientific research; asking: how to cope with potentially dead-end concepts? We saw that thinking about failure in science is not only beneficial if it leads directly toward future successes (e.g., as falsifications of a hypothesis; such as the classic Michelson and Morley experiment). Failures can also be fruitful if treated as failures in their own right, including ambiguous or unresolved cases, and without the need to prove or disprove a particular model – but as a heuristic tool to conceive of viable alternatives. The capacity to arrive at new insights and better models in science fundamentally hinges on the identification of choices with pursuit-worthy alternatives. But how to find promising options? One option is to treat a concept as a failure by taking it apart to probe the reasoning that fueled its conceptual foundation.

Let us to close with the *meta-value* of failure analysis. Historical and philosophical studies of scientific concepts can explain how scientific concepts developed, how their application changed, and how their embedding in an overarching framework would lead to different interpretations of the data today. Explicit attention to failure here concerns the issue of whether an idea has lost its heuristic power and is kept alive more as a remnant of a framework that itself should be reconsidered in its defining assumptions. Failure analysis consequently propels us to question philosophical intuitions about scientific concepts. Regularly, such intuitions do not reside in causal reasoning and are a product of historical growth.

Intellectual intuition, as a historical outcome, constitutes a non-negligible effect in the context of science communication and funding. In science communication, details and background are naturally shortened and omitted by appeal to conceptual analogs with greater explanatory intuition. Consequently, the layperson hears about science as a progressive, accumulative enterprise where older ideas lead to better ones – or are dropped. Failure, in this context, gets perceived as catastrophic, not a normal process of probing current understanding. However, all ideas constitute an intellectual expression of their time. So they routinely do not end up as failures because they were proven to be fraudulent or evidently false, or had no observations associated with them. Instead, they are gradually leaving active debate and practice that moved on to a different style of explanation under other frameworks by which to investigate phenomena.

Beyond the expert-layperson divide, we find similar rhetorical and explanatory strategies and shortcuts implemented in science-internal contexts, where they may not always be perceived directly, or where rhetorical maneuvers are not thought of as equally strong in their influence on judgments of theory and experiment. This is a critical misconception, as historical studies show. A case in point is Ceccarelli (2001) examination of the rhetoric strategies in three influential interdisciplinary works: Theodosius Dobzhansky’s *Genetics and the Origin of Species;* Erwin Schrödinger’s *What Is Life? The Physical Aspect of the Living Cell; and* Edward O. Wilson’s *Consilience: The Unity of Knowledge. –* The latter notably analyzed as a failure.

Science communication bridges not simply the layman-expert gap. It connects and disconnects practitioners in scientific research. The increasing specialization, even within a particular discipline such as neuroscience, implies a dilemma of multiple kinds of expertise. To communicate the highly technical nature of current research, scientists are encouraged to market its broader “impact” and “relevance” beyond an expert niche. Thus they link their studies to ideas more widely heard of, which have recognition value.

This has its price. Conceptual shortcuts, analogies, and metaphors – such as grandmother cells, next to countless other examples like maps and machines – constituted great tools throughout the history of science. But metaphorical thinking has its risks, endangering us to overlook other significant factors. As the cyberneticists Rosenblueth and Wiener cautioned: “The price for metaphor is eternal vigilance.” (Lewontin, 2001) Metaphors in science quickly develop a life of their own (a successful albeit hotly debated example is the notion of information from cybernetics that entered and transformed genetics; Kay, 2000). The peril of metaphors is the temptation to take them literally. The idea of the grandmother cell has been such a case.

In the end, there are multiple ways of dealing with failures in science. One option is to argue for the benefit of keeping contested ideas such as grandmother cells alive, even as an imperfect model of higher-level processing. Another, equally instructive option, outlined here, is to use them explicitly as failures to actively engage with our limits of understanding and probe for blind spots.

## Author Contributions

The author confirms being the sole contributor of this work and has approved it for publication.

## Conflict of Interest

The author declares that the research was conducted in the absence of any commercial or financial relationships that could be construed as a potential conflict of interest.

## References

[B1] Adrian E. D. Matthews R. (1927). The action of light on the eye. Part 1. the discharge of impulses in the optic nerve and its relation to the electric changes in the retina. *J. Physiol.* 97 378–414. 10.1113/jphysiol.1927.sp002410 PMC1514941 16993896

[B2] Bailer-Jones D. M. (2002). “Models, metaphors and analogies,” in *The Blackwell Guide to the Philosophy of Science 114*, eds Machamer P. Silberstein M., (Malden, MA: Blackwell Publishers Ltd.).

[B3] Barlow H. B. (1972). Single units and sensation: a neuron doctrine for perceptual psychology? *Perception* 1 371–394. 10.1068/p010371 4377168

[B4] Barlow H. B. (1995). “The neuron in perception,” in *The Cognitive Neurosciences*, ed. Gazzaniga M. S., (Cambridge, MA: MIT Press), 415–434.

[B5] Barlow H. B. (2009). “Grandmother cells, symmetry, and invariance: how the term arose and what the facts suggest,” in *The Cognitive Neurosciences*, 4th Edn, ed. Gazzaniga M., (Cambridge, MA: MIT Press), 309–320.

[B6] Barnes B. (1982). On the extensions of concepts and the growth of knowledge. *Sociol. Rev.* 30 23–44. 10.1111/j.1467-954x.1982.tb00652.x

[B7] Barwich A. S. (2013). A pluralist approach to extension: the role of materiality in scientific practice for the reference of natural kind terms. *Biol. Theory* 7 100–108. 10.1007/s13752-012-0083-x

[B8] Barwich A. S. (2018a). How to be rational about empirical success in ongoing science: the case of the quantum nose and its critics. *Stud. Hist. Philos. Biol. Biomed. Sci.* 69 40–51. 10.1016/j.shpsa.2018.02.005 29857800

[B9] Barwich A. S. (2018b). “Measuring the world: towards a process model of perception,” in *Everything Flows: Towards a Processual Philosophy of Biology*, eds Nicholson D. Dupre J., (Oxford: Oxford University Press), 337–356.

[B10] Barwich A. S. Bschir K. (2017). The manipulability of what? the history of g-protein coupled receptors. *Biol. Philos.* 32 1317–1339. 10.1007/s10539-017-9608-9

[B11] Bickle J. (2015). Marr and reductionism. *Top. Cogn. Sci.* 7 299–311. 10.1111/tops.12134 25772159

[B12] Blakemore C. Cooper G. F. (1970). “Development of the brain depends on the visual environment. *Nature* 228 477–478. 10.1038/228477a0 5482506

[B13] Bowers J. S. (2009). On the biological plausibility of grandmother cells: implications for neural network theories in psychology and neuroscience. *Psychol. Rev.* 116 220–251. 10.1037/a0014462 19159155

[B14] Burnston D. C. (2016). A contextualist approach to functional localization in the brain. *Biol. Philos.* 31 527–550. 10.1007/s10539-016-9526-2

[B15] Ceccarelli L. (2001). *Shaping Science With Rhetoric: The Cases of Dobzhansky, Schrodinger, and Wilson.* Chicago: University of Chicago Press.

[B16] Chang H. (2004). *Inventing Temperature: Measurement and Scientific Progress.* Oxford: Oxford University Press.

[B17] Chang H. (2012). *Is Water H2O? Evidence, Realism and Pluralism.* Dordrecht: Springer Science & Business Media.

[B18] Chemero A. (2009). *Radical Embodied Cognitive Science.* Cambridge, MA: MIT Press.

[B19] Collins A. M. Quillian M. R. (1969). Retrieval time from semantic memory. *J. Verbal Learning Verbal Behav.* 8 240–247. 10.1016/s0022-5371(69)80069-1

[B20] Coltheart M. (2016). Grandmother cells and the distinction between local and distributed representation. *Lang. Cogn. Neurosci.* 23 350–358. 10.1080/23273798.2016.1232420

[B21] Craver C. F. (2007). *Explaining the Brain: Mechanisms and The Mosaic Unity of Neuroscience.* Oxford: Oxford University Press.

[B22] English K. (1998). Understanding science: when metaphors become terms. *ASP* 19-22 151–163. 10.4000/asp.2800

[B23] Firestein S. (2012). *Ignorance: How It Drives Science.* New York: Oxford University Press.

[B24] Firestein S. (2015). *Failure: Why Science Is So Successful.* New York: Oxford University Press.

[B25] Fodor J. A. (1985). Precis of the modularity of mind. *Behav. Brain Sci.* 8 1–5. 10.1017/S0140525X15000631 26077688

[B26] Gaschler K. (2006). One person, one neuron? *Sci. Am. Mind* 17 76–82. 10.1038/scientificamericanmind0206-76

[B27] Gazzaniga M. Ivry R. B. (eds) (2013). *Cognitive Neuroscience: The Biology of the Mind*, 4th Edn New York, NY: W.W. Norton.

[B28] Gefter A. (2015). *The Man Who Tried to Redeem The World with Logic. Nautilus 21.* Available at: http://nautil.us/issue/21/information/the-man-who-tried-to-redeem-the-world-with-logic (accessed July 2, 2019).

[B29] Glennerster A. (2007). Marr’s vision: Twenty-five years on. *Curr. Biol.* 17 R397–R399. 17550760 10.1016/j.cub.2007.03.035

[B30] Gosline A. (2005). *Why Your Brain Has a ‘Jennifer Aniston Cell. New Scientist.* Available at: https://www.newscientist.com/article/dn7567-why-your-brain-has-a-jennifer-aniston-cell/ (accessed March 15, 2017).

[B31] Gross C. G. (1998). *Brain, Vision, Memory: Tales in the History of Neuroscience.* Cambridge, MA: MIT Press.

[B32] Gross C. G. (2002). Genealogy of the “grandmother cell”. *Neuroscientist* 8 512–518. 10.1016/j.meegid.2016.04.006 12374433

[B33] Grünbaum A. (1976). Ad hoc auxiliary hypotheses and falsificationism. *Br. J. Philos. Sci.* 27 329–362. 10.3758/s13423-018-1488-8 29799092

[B34] Hesse M. B. (1966). *Models and Analogies in Science.* Southbend, IN: University of Notre Dame Press.

[B35] Hohwy J. (2013). *The Predictive Mind.* Oxford: Oxford University Press.

[B36] Holcombe A. O. (2009). “The binding problem,” in *The Sage Encyclopedia of Perception*, ed. Bruce Goldstein E., (Thousand Oaks, CA: Sage).

[B37] Horton J. C. Adams D. L. (2005). The cortical column: a structure without a function. *Philos. Trans. R. Soc. Lond. B Biol. Sci.* 360 837–862. 10.1098/rstb.2005.1623 15937015 PMC1569491

[B38] Hubel D. (1988). *Eye, Brain, and Vision.* Armonk, NY: Scientific American Library.

[B39] Hubel D. H. Wiesel T. N. (1962). Receptive fields, binocular interaction and functional architecture in cats visual cortex. *J. Physiol.* 160 106–154. 10.1113/jphysiol.1962.sp006837 14449617 PMC1359523

[B40] Hubel D. H. Wiesel T. N. (1963). Receptive fields of cells in striate cortex of very young, visually inexperienced kittens. *J. Neurophysiol.* 26 994–1002. 10.1152/jn.1963.26.6.994 14084171

[B41] Hubel D. H. Wiesel T. N. (1965). Receptive fields and functional architecture in two nonstriate visual areas (18 and 19) of the cat. *J. Neurophysiol.* 28 229–289. 10.1152/jn.1965.28.2.229 14283058

[B42] Hubel D. H. Wiesel T. N. (1979). Brain mechanisms of vision. *Sci. Am.* 241 40–49. 91195 10.1038/scientificamerican0979-150

[B43] Kay L. E. (2000). *Who Wrote the Book of Life? A History of the Genetic Code.* Stanford: Stanford University Press.

[B44] Keijzer F. (2001). *Representation and Behavior.* Cambridge, MA: MIT Press.

[B45] Kellert S. H. Longino H. E. Waters C. K. (eds) (2006). *Scientific pluralism.* Minneapolis: University of Minnesota Press.

[B46] Khamsi R. (2005). Jennifer aniston strikes a nerve. *Nat. News* 10.1038/news050620-7 (accessed October 15, 2019).

[B47] Konorski J. (1967). *Integrative Activity of the Brain: An Interdisciplinary Approach.* Chicago: University of Chicago.

[B48] Laurent G. (2002). Olfactory network dynamics and the coding of multidimensional signals. *Nat. Rev. Neurosci.* 3:884. 10.1038/nrn964 12415296

[B49] Lewontin R. C. (2001). *The Triple Helix: Gene, Organism, and Environment.* Cambridge, MA: Harvard University Press.

[B50] Livingstone M. S. Vincent J. L. Arcaro M. J. Srihasam K. Schade P. F. Savage T. (2017). Development of the macaque face-patch system. *Nat. Commun.* 8:14897. 10.1038/ncomms14897 28361890 PMC5381009

[B51] Longino H. (2019). *The Social Dimensions of Scientific Knowledge. In: Stanford Encyclopedia of Philosophy, ed. by Ed Zalta.* Available at: https://plato.stanford.edu/entries/scientific-knowledge-social/ (accessed July 6, 2019).

[B52] Machamer P. Darden L. Craver C. F. (2000). Thinking about mechanisms. *Philos. Sci.* 67 1–25.

[B53] Marr D. (1982). *Vision. A computational Investigation into the Human Representation An Processing of Visual Information.* Cambridge, MA: MIT Press.

[B54] McClelland J. L. McNaughton B. L. O’Reilly R. C. (1995). Why there are complementary learning systems in the hippocampus and neocortex: insights from the successes and failures of connectionist models of learning and memory. *Psychol. Rev.* 102:419. 10.1037/0033-295X.102.3.419 7624455

[B55] McCulloch W. S. Pitts W. (1943). A logical calculus of the ideas immanent in nervous activity. *Bull. Math. Biophys.* 5 115–133. 10.1007/bf02478259 2185863

[B56] Medarwar P. (1959/1999). “Is the scientific paper a fraud?,” in *Communicating Science: Professional Contexts*, eds Scanlon E. Hill R. Junker K., (London: Routledge), 27–31.

[B57] Mishkin M. (1966). “Visual mechanisms beyond the striate cortex,” in *Frontiers in Physiological Psychology*, ed. Russel R. W., (New York, NY: Academic Press), 93–119.

[B58] Morgan M. S. Norton M. N. (2017). Narrative science and narrative knowing. Introduction to special issue on narrative science. *Stud. Hist. Philos. Sci.* 62 1–5. 10.1016/j.shpsa.2017.03.005 28583354

[B59] Mountcastle V. B. (1957). Modality and topographic properties of single neurons of cat’s somatic sensorycortex. *J. Neurophysiol.* 20 408–434. 10.1152/jn.1957.20.4.408 13439410

[B60] Neisser U. (1976). *Cognition and Reality.* New York, NY: WH Freeman.

[B61] Newell A. Simon H. (1976). Computer science as empirical inquiry: symbols and search. *Commun. ACM* 1 113–126. 10.1145/360018.360022

[B62] O’Malley M. A. Elliott K. C. Haufe C. Burian R. M. (2009). Philosophies of funding. *Cell* 138 611–615. 10.1016/j.cell.2009.08.008 19703386

[B63] Oram M. W. Perrett D. I. (1994). Modeling visual recognition from neurobiological constraints. *Neural Netw.* 7 945–972. 10.1016/s0893-6080(05)80153-4

[B64] Perrett D. I. Mistlin A. J. Chitty A. J. (1987). Visual neurones responsive to faces. *Trends Neurosci.* 10 358–364. 10.1016/0166-2236(87)90071-3

[B65] Perrett D. I. Rolls E. T. Caan W. (1982). Visual neurons responsive to faces in the monkey temporal cortex. *Exp. Brain Res.* 47 329–342. 7128705 10.1007/BF00239352

[B66] Piccinini G. (2004). The first computational theory of mind and brain: a close look at mcculloch and pitts’s ‘logical calculus of ideas immanent in nervous activity. *Synthese* 141 175–215. 10.1023/b:synt.0000043018.52445.3e

[B67] Plaut D. C. McClelland J. L. (2010). Locating object knowledge in the brain: comment on bowers’s (2009) attempt to revive the grandmother cell hypothesis. *Psychol. Rev.* 117 284–288. 10.1037/a0017101 20063976

[B68] Poldrack R. A. (2006). Can cognitive processes be inferred from neuroimaging data? *Trends Cogn. Sci.* 10 59–63. 10.1016/j.tics.2005.12.004 16406760

[B69] Poldrack R. A. (2011). Inferring mental states from neuroimaging data: from reverse inference to large-scale decoding. *Neuron* 72 692–697. 10.1016/j.neuron.2011.11.001 22153367 PMC3240863

[B70] Pribram K. H. Mishkin M. (1955). Simultaneous and successive visual discrimination by monkeys with inferotemporal lesions. *J. Comp. Physiol. Psychol.* 48 198–202. 10.1037/h0049140 13242690

[B71] Quiroga R. Q. Alexander K. Koch C. Fried I. (2009). Explicit encoding of multimodal percepts by single neurons in the human brain. *Curr. Biol.* 19 1308–1313. 10.1016/j.cub.2009.06.060 19631538 PMC3032396

[B72] Quiroga R. Q. Fried I. Koch C. (2013). Brain cells for grandmother. *Sci. Am.* 308 30–35. 10.1038/scientificamerican0213-30 23367781

[B73] Quiroga R. Q. Kraskov A. Mormann F. Fried I. Koch C. (2014). Single-cell responses to face adaptation in the human medial temporal lobe. *Neuron* 84 363–369. 10.1016/j.neuron.2014.09.006 25263754 PMC4210637

[B74] Quiroga R. Q. Kreiman G. Koch C. Fried I. (2008). Sparse but not ‘grandmother-cell’ coding in the medial temporal lobe. *Trends Cogn. Sci.* 12 87–91. 10.1016/j.tics.2007.12.003 18262826

[B75] Quiroga R. Q. Reddy L. Kreiman G. Koch C. Fried I. (2005). Invariant visual representation by single neurons in the human brain. *Nature* 435:1102. 10.1038/nature03687 15973409

[B76] Reddy L. Thorpe S. J. (2014). Concept cells through associative learning of high-level representations. *Neuron* 84 248–251. 10.1016/j.neuron.2014.10.004 25374351

[B77] Rips L. J. Shoben E. J. Smith E. E. (1973). Semantic distance and the verification of semantic relations. *J. Verbal Learning Verbal Behav.* 12 1–20. 10.1016/s0022-5371(73)80056-8

[B78] Rolls E. T. (1984). Neurons in the cortex of the temporal lobe and in the amygdala of the monkey with responses selective for faces. *Hum. Neurobiol.* 3 209–222. 6526707

[B79] Root-Bernstein R. S. (1988). Setting the stage for discovery. *Sciences* 28 26–34. 10.1002/j.2326-1951.1988.tb03019.x

[B80] Root-Bernstein R. S. (1989). How scientists really think. *Perspect. Biol. Med.* 32 472–488. 10.1353/pbm.1989.0017 2674885

[B81] Rose D. (1996). Some reflections on (or by?) grandmother cells. Guest editorial. *Perception* 25 881–886. 10.1068/p250881 8938002

[B82] Roskies A. L. (1999). The binding problem. *Neuron* 24 7–9. 10677022 10.1016/s0896-6273(00)80817-x

[B83] Roy A. (2012). A theory of the brain: localist representation is used widely in the brain. *Front. Psychol.* 3:551. 10.3389/fpsyg.2012.00551 23426117 PMC3576056

[B84] Roy A. (2013). An extension of the localist representation theory: grandmother cells are also widely used in the brain. *Front. Psychol.* 4:300. 10.3389/fpsyg.2013.00300 23745119 PMC3662881

[B85] Roy A. (2017). The theory of localist representation and of a purely abstract cognitive system: the evidence from cortical columns, category cells, and multisensory neurons. *Front. Psychol.* 8:186. 10.3389/fpsyg.2017.00186 28261127 PMC5311062

[B86] Schickore J. (2018). Explication work for science and philosophy. *J. Philos. Hist.* 12 191–211. 10.1163/18722636-12341387 24987787

[B87] Schickore J. (in review). *“Is “Failing Well” A Sign of Scientific Virtue?”.*

[B88] Sejnowski T. C. (2014). “Grandmother cells,” in *What Idea If Ready For Retirement?*, ed. Brockman J., (Millbank: Edge Foundation Inc.).

[B89] Shepherd G. M. (2009). *Creating Modern Neuroscience: The Revolutionary 1950s.* Oxford: Oxford University Press.

[B90] Stanford P. K. (2006). *Exceeding Our Grasp: Science, History, and the Problem of Unconceived Alternatives.* Oxford: Oxford University Press.

[B91] Temmerman R. (1995). The process of revitalisation of old words: ‘Splicing’, a case study in the extension of reference. *Terminology* 2 107–128. 10.1075/term.2.1.06tem

[B92] Temmerman R. (2000). *Towards New Ways of Terminology Description: The Sociocognitive Approach.* Amsterdam: Benjamins.

[B93] Treisman A. (1996). The binding problem. *Curr. Opin. Neurobiol.* 6 171–178. 8725958 10.1016/s0959-4388(96)80070-5

[B94] Van Gelder T. (1995). What might cognition be, if not computation? *J. Philos.* 92 345–381. 10.2307/2941061

[B95] Yamane S. Kaji S. Kawano K. (1988). What facial features activate face neurons in the inferotemporal cortex of the monkey? *Exp. Brain Res.* 73 209–214. 10.1007/bf00279674 3208858

[B96] Zimmer C. (2009). *The Legend of Grandmother Cells Continues.” Discovery Magazine.* Available at: http://blogs.discovermagazine.com/loom/2009/07/23/the-legend-of-grandmother-cells-continues/ (accessed July 2, 2019).

